# Motor Skills of Children and Adolescents Are Influenced by Growing up Barefoot or Shod

**DOI:** 10.3389/fped.2018.00115

**Published:** 2018-04-25

**Authors:** Astrid Zech, Ranel Venter, Johanna E. de Villiers, Susanne Sehner, Karl Wegscheider, Karsten Hollander

**Affiliations:** ^1^Institute of Sports Science, University of Jena, Jena, Germany; ^2^Department of Sport Science, Stellenbosch University, Stellenbosch, South Africa; ^3^Institute of Medical Biometry and Epidemiology, University Medical Center Hamburg-Eppendorf, Hamburg, Germany; ^4^Department of Sports and Exercise Medicine, Institute of Human Movement Science, University of Hamburg, Hamburg, Germany; ^5^Department of Sports and Rehabilitation Medicine, BG Trauma Hospital of Hamburg, Hamburg, Germany

**Keywords:** motor performance, barefoot, balance, jump, sprint

## Abstract

**Background:** The objective of this study was to evaluate the association between growing up barefoot or shod and the development of motor performance during childhood and adolescence.

**Methods:** Habitual barefoot and shod children and adolescents between 6 and 18 years were recruited in South Africa and Germany. Participants completed balance, standing long jump and 20 m sprint tests in barefoot and shod conditions. Outcomes were analyzed in separate mixed-effects linear regressions for three age groups according to stages of development (6–10, 11–14, and 15–18 years). All models were adjusted for confounders: sex, ethnicity, BMI, PAQ score and order of tests (barefoot vs. shod).

**Results:** Three hundred and eight-five habitually barefoot and 425 habitually shod children participated. Significant age by footwear effects were found for the jump (*p* = 0.032) and sprint test (*p* = 0.041). Habitually barefoot children aged 6–10 years scored higher in the balance test (*p* = 0.015) and standing long jump (*p* = 0.005) whereas habitually shod children sprinted faster (*p* < 0.001). Faster sprint times were found for habitually shod participants between 11 and 14 years (*p* < 0.001). Habitually barefoot adolescents between 15 and 18 years of age showed a greater long jump distance (*p* < 0.001) but slower sprint times (*p* = 0.014) than shod adolescents.

**Conclusions:** The results emphasize the importance of footwear habits for the development of motor skills during childhood and adolescence. Regular physical activities without footwear may be beneficial for the development of jumping and balance skills, especially in the age of 6 to 10 years.

## Introduction

Improvements of motor skills are basic processes of growth, maturation and development during childhood and adolescence. They depend on permanent interactions between the neuromuscular system and environment and can be influenced by multiple external and internal factors [1]. These include anthropometrical and muscular characteristics of the foot. It has been shown [2–4] that different structural foot types such as high and low arches can influence the performance of basic motor skills such as balancing, jumping or sprinting. Since the foot morphology differs between barefoot and shod populations [5, 6], footwear habits have long been discussed to play an important role for motor learning and motor control [7]. Evidence exists that compared to shod conditions, barefoot situations directly change gait biomechanics, postural control and jumping movements in children and adults [8–14]. Individuals growing up barefoot also tend to have wider feet with a higher arch and smaller hallux angles [7]. This emphasizes the hypothesis that the development of basic motor skills during childhood and adolescence partly depends on the amount of physical activity under barefoot conditions. However, to our knowledge, no study has examined the potential relationship between regular barefoot activities and motor skills. A comparison of motor skills between habitually barefoot and shod individuals in the same setting is difficult, since barefoot habits seem to be influenced by sociocultural and regional factors [7, 15]. Individuals in western countries are used to wear shoes during almost all outdoor activities whereas especially children in developmental countries are mostly seen barefoot. It is therefore not surprising that existing studies regarding footwear habits and foot characteristics or running biomechanics compared habitual barefoot and shod populations of different regions/countries [5, 7].

The objective of this was to evaluate, for the first time, the potential interaction between growing up barefoot or shod and performance in basic motor competencies during the different stages of childhood and adolescence.

## Materials and methods

A cross-sectional observational study was conducted in South Africa and Germany between March 2015 and June 2016 [16]. Children and adolescents between 6 and 18 years were recruited in schools across rural and urban areas in the Western Cape, in South Africa and Northern Germany. The two populations were chosen due to their different footwear habits. Whereas South African children are generally used to walk barefoot during the day almost all German children wear shoes during school time and for most of recreational activities. The recruitment of participants took place in primary and secondary schools with no restriction to school type. In South Africa, primary school attendees are aged between 6 and 13/14 years and secondary school children between 13/14 and 18 years. In Germany, children in primary school are aged 6 to 9/10 years and in secondary school 9/10 to 18 years. After approval from the German and South African supervisory school authorities, schools were randomly selected per stratum (representing a combination of district and type of school) and contacted via email by the principal investigators.

Inclusion criteria were an age between 6 and 18 years, regular attendance in physical education classes at the time of testing and physical activity for at least 120 accumulative minutes per week (parent reported). Exclusion criteria were current injuries and orthopedic, neurological or neuromuscular conditions that may influence motor performance. A minimum of daily activities without footwear was required to be classified as habitually barefoot: (A) during school and in and around the house or (B) during sports/recreational activities and in and around the house [16]. Ethical approval has been obtained from local ethics committees in South Africa (protocol number HS1153/2014) and Germany (protocol number PV4971). Children with a signed parental consent were tested during their regular physical education classes at school by a team of investigators.

Prior to the testing, all participants were assessed for height and weight. The BMI was calculated by using the formula weight/height^2^. Additionally, the self-reported physical activity status was assessed using the Physical Activity Questionnaire for children (PAQ-C) and adolescents (PAQ-A). Basic motor competencies involving lower extremities were tested in barefoot and shod conditions. Participants completed a balance, standing long jump and flying 20 m sprint test.

### Balance test

A standardized backward balance task suitable for children between 6 and 18 years of age [17] was used to assess dynamic postural control. Participants were instructed to walk backwards in a self-selected, comfortable speed over three balance beams of 6, 4.5, and 3 cm width. Excluding the first step, the number of subsequent successful steps was counted on each beam. The test on each beam finished if balance was lost and one foot touched the ground or a maximum of eight steps was achieved. During balancing, the children were asked to look straight forward on a fixed point placed on the wall at eye level and to put their next step directly behind the other foot. For analysis, the scores of two trials on each of the three beams were added to a total score per condition (maximum 48 points).

### Jump test

For the standing long jump test [18] participants stood behind a labeled take-off line and were asked to jump as far as possible. The distance between the start and landing point (back of the heal nearest to the take-off line) was read off and recorded manually. The test was repeated three times per footwear condition and the distance of the best trial was used for analysis.

### Sprint test

Maximum sprint time was assessed using the flying 20-m sprint test. Time was recorded using a magnetic sensor system with magnetic gates (SmarTracks Diagnostics, Humotion, Germany) in Germany and speed gates (Brower Timing Systems, USA) in South Africa.

Outcomes were analyzed in separate mixed-effects linear regressions for three age groups according to stages of development (6–10, 11–14, and 15–18 years). Barefoot and shod data were included and marginal means were calculated. All models were adjusted for confounders sex, ethnicity, BMI, PAQ score and order of tests (barefoot vs. shod). The confounders were additionally adjusted for age.

## Results

The final sample consisted of 810 children and adolescents from 22 primary and secondary schools. Habitual barefoot children (*n* = 385, 49.4% female) had a mean (SD) age of 11.5 (3.2) years, BMI of 19.55 (3.94) and PAQ score of 3.06 (0.66). Habitual shod children (*n* = 425, 50.6% female) had a mean (SD) age of 12.4 (3.4) years, BMI of 19.33 (3.88), and PAQ score of 2.75 (0.66). Descriptive data of motor skills in the three age groups and for both test conditions (barefoot vs. shod) are shown in Table 1. In all outcomes, sex (*p* < 0.001), BMI (*p* < 0.001), and PAQ score (balance: *p* < 0.001, long jump: *p* = 0.033, sprint: *p* = 0.001) were significant confounders. The standing long jump was further influenced by ethnicity (*p* = 0.002) (Supplementary Images 1–3).

**Table 1:** Descriptive data of balance, long jump, and sprint performance for habitually barefoot and shod individuals divided by age groups and both test conditions (barefoot vs. shod testing).

		**Balance (sum score)**		**Long jump (cm)**		**Sprint time (s)**	
**Test condition**		**Barefoot**	**Shod**	**Barefoot**	**Shod**	**Barefoot**	**Shod**
**HABITUALLY BAREFOOT INDIVIDUALS**							
6–10 years	Mean (SD)	30.0 (9.4)	31.8 (10.4)	128.9 (20.7)	128.6 (20.8)	4.43 (0.51)	4.58 (0.53)
	*N*	94	81	122	80	121	82
11–14 years	Mean (SD)	32.5 (8.8)	35.5 (8.5)	152.8 (23.9)	152 (25.1)	3.94 (0.36)	3.61 (0.38)
	*N*	135	118	153	118	153	118
15–18 years	Mean (SD)	37.7 (8.3)	38.1 (8.2)	184.8 (31.4)	183.0 (35.2)	3.61 (0.38)	3.66 (0.43)
	*N*	108	90	108	90	105	86
All	Mean (SD)	33.5 (9.3)	35.1 (9.3)	154.2 (33.3)	155.2 (34.7)	4.01 (0.53)	4.07 (0.57)
	*N*	337	289	383	288	379	286
**HABITUALLY SHOD INDIVIDUALS**							
6–10 years	Mean (SD)	26.8 (8.7)	30.5 (9.2)	126.0 (18.9)	124.7 (17.2)	4.10 (0.74)	4.15 (0.62)
	*N*	100	99	100	99	94	94
11–14 years	Mean (SD)	30.5 (9.6)	34.4 (9.3)	151.6 (25.2)	150.3 (24.8)	3.80 (0.41)	3.78 (0.48)
	*N*	155	154	154	153	133	132
15–18 years	Mean (SD)	34.7 (9.4)	38.5 (8.6)	169.0 (29.5)	171.9 (30.2)	3.62 (0.36)	3.59 (0.32)
	*N*	169	169	168	168	159	159
All	Mean (SD)	31.29 (9.8)	35.13 (9.5)	152.5 (30.6)	152.9 (31.5)	3.79 (0.53)	3.79 (0.51)
	*N*	424	422	422	420	382	383

**Figure 1:**
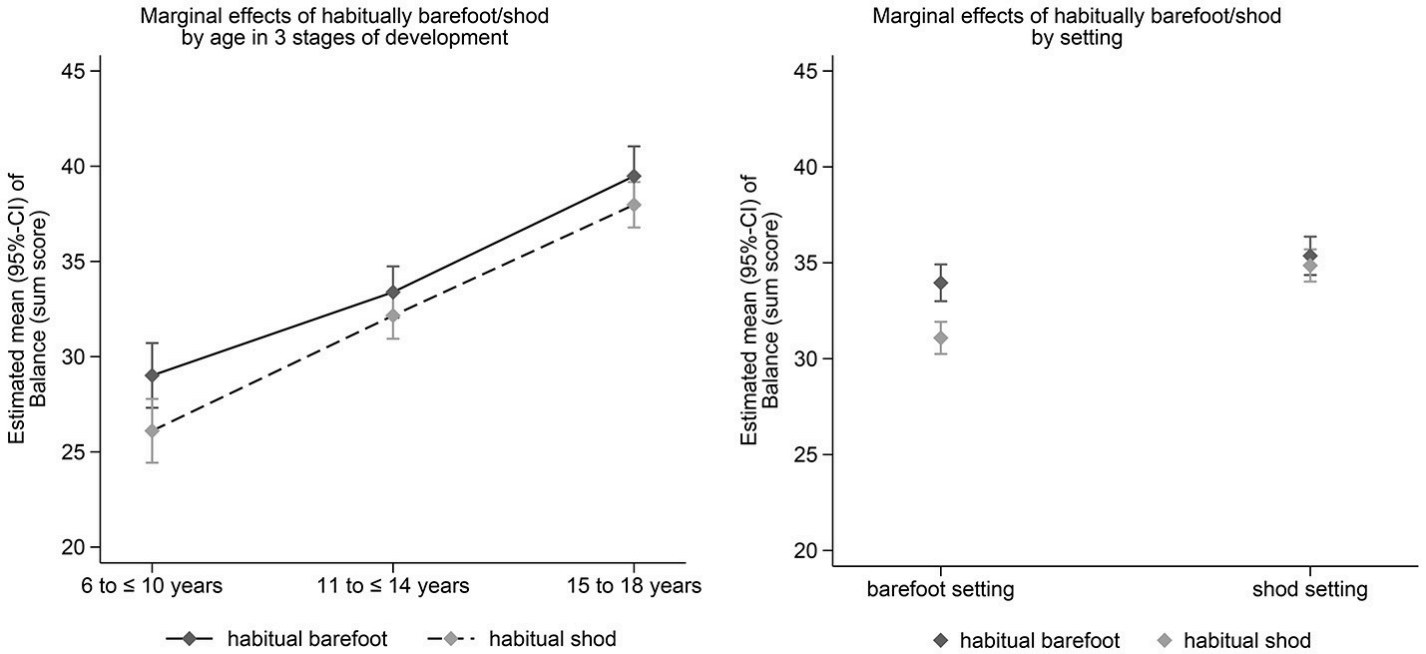
Estimated marginal effects (95%-CI) of habitual footwear use on balance performance in the three age groups (left) and for the setting (barefoot vs. shod testing, right) after adjustment for confounders.

**Figure 2:**
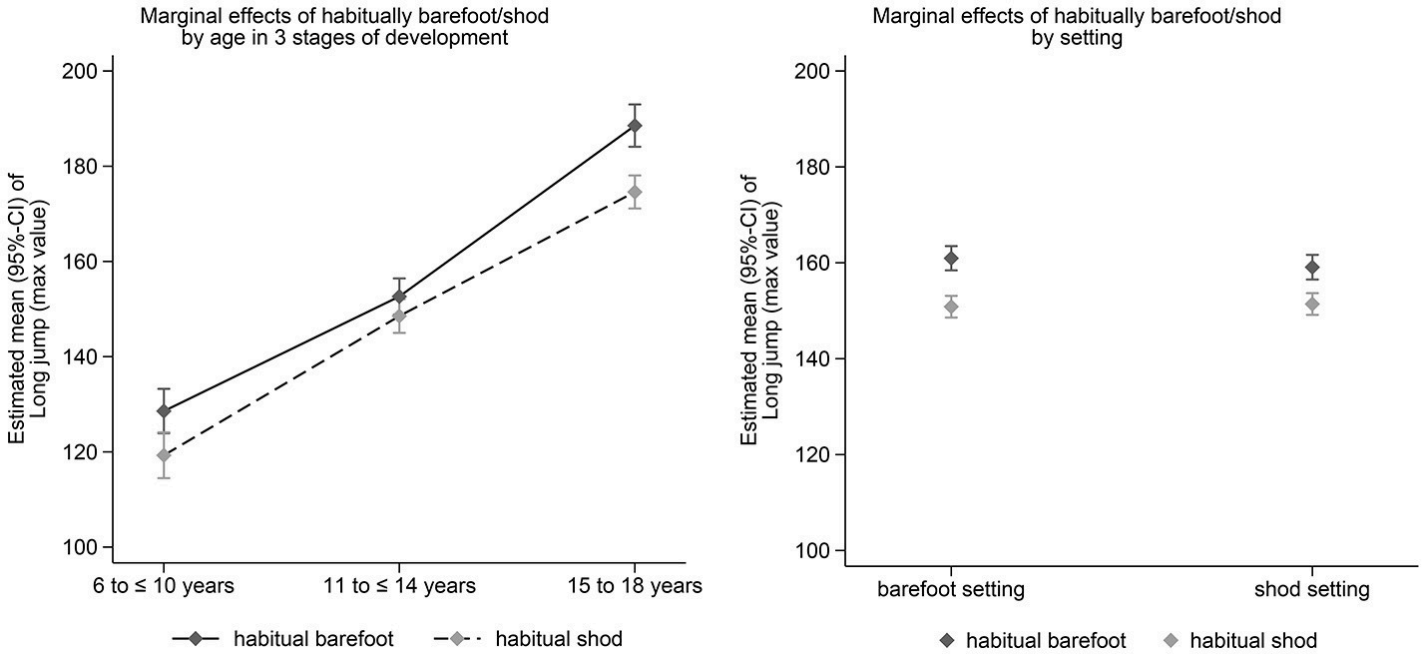
Estimated marginal effects (95%-CI) of habitual footwear use on standing long jump performance in the three age groups (left) and for the setting (barefoot vs. shod testing, right) after adjustment for confounders.

**Figure 3:**
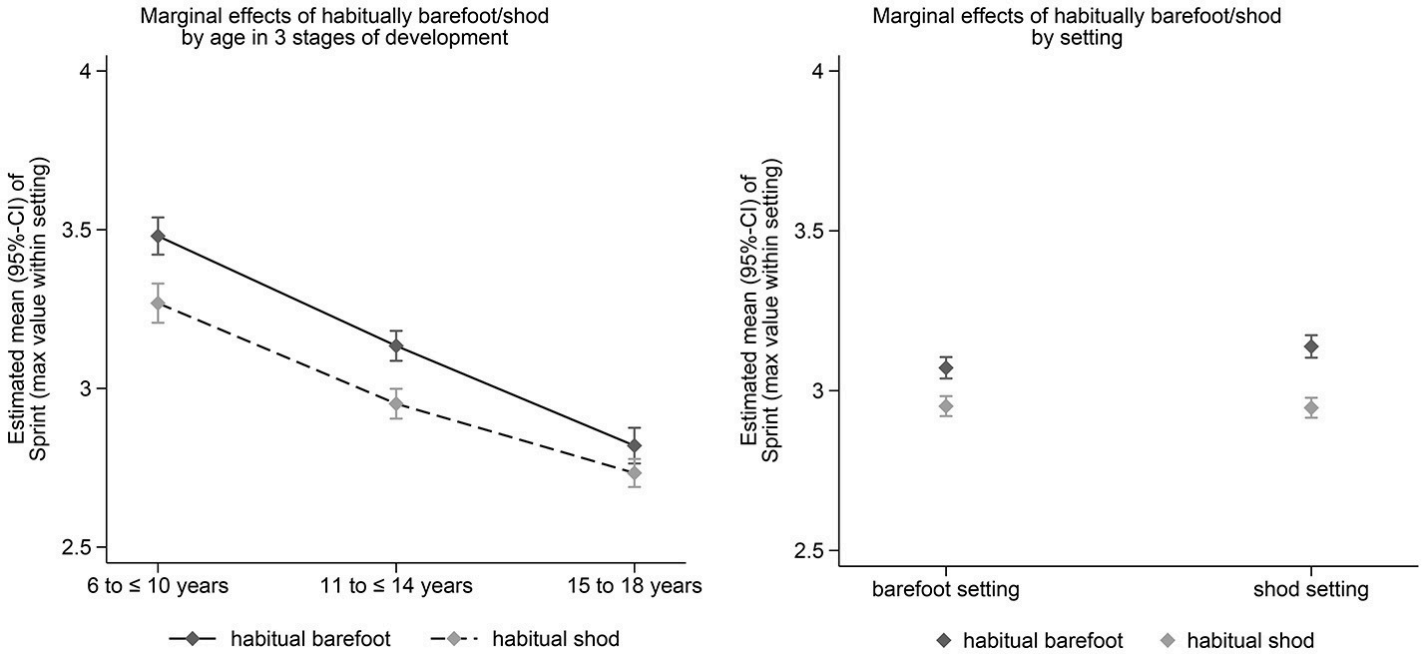
Estimated marginal effects (95%-CI) of habitual footwear use on sprint performance in the three age groups (left) and for the setting (barefoot vs. shod testing, right) after adjustment for confounders.

Adjusted estimated marginal effects (95%-CI) of habitual footwear use on balance, long jump and sprint performance in the three age groups (left) and for the setting (barefoot vs. shod testing, right) after adjustment for confounders are shown in Figures 1–3. Significant effects of habitual footwear use were found for the standing long jump (*p* = 0.032) and sprint test (*p* = 0.041). Post-hoc comparisons within age groups showed that habitual barefoot children aged 6–10 years scored higher in the backward balance test (*p* = 0.015) and standing long jump (*p* = 0.005) whereas shod children were faster in the sprint test (*p* < 0.001). Faster sprint times were also found for habitual shod participants between 11 and 14 years (*p* < 0.001). Barefoot adolescents between 15 and 18 years of age showed a greater long jump distance (*p* < 0.001) but slower sprint times (*p* = 0.014) than shod adolescents.

Significant interactions were also found for test conditions and habitual footwear use. In the barefoot population, the performance in all motor skills was significantly different between barefoot and shod test conditions (balance: *p* = 0.001, long jump: *p* = 0.002, sprint: *p* < 0.001) whereas the shod population had less differences (balance: *p* < 0.001).

## Discussion

The main finding of this study was that motor performance differs between habitually barefoot and shod children and adolescents. Regular barefoot activities during childhood seem to be beneficial for the development of balance and jumping skills whereas shod individuals show better sprinting skills. Fewer differences were observed during adolescence although there are greater jump distances and slower sprint times in barefoot individuals. The differences between both populations in the balance and jumping performance were particularly influenced by the barefoot test condition whereas the shod test condition alone revealed no effects of habitual footwear use. To the best of our knowledge, this is the first study investigating the relevance of growing up under different footwear conditions for motor performance.

Considering previous studies, some speculations can be made regarding underlying mechanisms for this finding. A likely explanation is that footwear habits influence the musculoskeletal architecture of the foot [6, 7] which in turn may be associated with motor performance [2, 3]. Hertel and coauthors [2] and Tsai et al. [3] showed that standing balance control differs between healthy adults with different foot types. An association between jumping performance and foot anthropometrics has been reported by van Werkhoven and Piazza [4]. They showed that the best jumpers had longer lateral heel lengths and longer toes. Other studies [19, 20] however, did not observe any relationships between the foot arch structure and the performance in sprinting, jumping and balancing. Another possible reason for the differences in motor skills between both populations may be different adaptations of muscle strength to regular physical activities with or without footwear [19, 21, 22]. One study [23] showed that foot muscle strength is associated with sprinting and jumping skills in preschool-age children. Morita et al. [19] reported that sprinting and jumping performances of elementary school children are more related to toe flexor strength than to the foot arch. Furthermore, Zhang et al. [24] reported a larger size of the abductor hallucis, flexor digitorum brevis, and longus muscles in recreational runners with overpronated feet compared to runners with normal feet. The authors concluded that the morphological characteristics of foot muscles could affect foot kinematics and discussed a potential association with the risk of injuries. Another explanation could be that different biomechanics have influenced the motor performance in both populations. During running, habitual barefoot individuals tend to have different foot, ankle and knee kinematics as well as peak impact forces during landing than shod individuals [6–8]. Numerous studies also reported immediate changes in biomechanics when habitual shod individuals participate in barefoot activities [8–12, 25].

The tests used in this study are valid and reliable measures of basic motor skills in children and adolescent, and positively associated with health-related fitness in youth [26]. In agreement with previous literature, [17, 18] all tested motor skills improved between age 6 and 18 years. However, our results show that motor skill competencies of shod and barefoot individuals may develop differently during childhood and adolescence. Whereas barefoot children between ages 6 and 10 years scored higher in the backward balance test compared to shod children, no differences were found in adolescent participants. The early childhood years are fundamental for the development of balance and rapid improvements can be observed until the age of 9–10 years [17, 27]. This may explain the higher adaptability of balance control during this period to changing external conditions such as barefoot activities. In agreement with the smaller improvements in balance control during adolescence [17] the beneficial barefoot effects seem to become smaller at this age.

Habitual barefoot individuals generally reached greater jump distances than shod individuals although significant effects were found only for the age groups 6–10 and 15–18 years. The jumping performance of children is considerably influenced by foot muscle strength [23]. There is broad evidence for the influence of other lower extremity muscles (e.g., quadriceps femoris or calf muscles) [28, 29]. However, no study investigated the relationship between muscle strength and standing long jump performance in preadolescent children.

Although the jump performance is generally associated with the sprint performance, our study found slower sprint times in barefoot children in all age groups [30]. This may be due to differences in the physical condition or technical skills between both populations but also could be explained with the different settings. More than the balance or jumping tests, the sprinting test can be influenced by environmental factors such as the running surface, temperature or wind situations. All children were tested where their physical education lessons were held. Thus, habitually shod children were tested indoor in local sports halls whereas the habitually barefoot children were tested in sports halls, in school assembly halls or outdoors on different surfaces. The investigators in our study had no influence on this since most barefoot children were recruited in rural areas without standardized running tracks or sports halls.

The regional difference between both settings is the biggest limitation in this study. Barefoot children were exclusively recruited in South Africa and shod children in Northern Germany. Since habitual footwear use is influenced by sociocultural and climatic factors, [7] a recruitment of both populations within one setting appeared difficult. South African children walk barefoot most time of the day whereas German children are obligated to wear shoes at least during school time. In order to address methodological issues caused by the selective recruitment in different countries, the ethnic background and physical activity was included in the statistical model as confounding factors.

In conclusion, the results emphasize the importance of footwear habits for the development of motor skills during childhood and adolescence. This indicates that regular physical activities without footwear may be beneficial for the development of jumping and balance skills, especially in the age of 6–10 years. In order to evaluate the potential benefits but also risk factors of barefoot activities during childhood further research using a prospective design is needed.

## Ethics statement

Ethical approval has been obtained from Stellenbosch University ethics committee in South Africa (protocol number HS1153/2014) and the ethics committee of the medical association Hamburg, Germany (protocol number PV4971). Children with a signed parental consent were tested.

## Author contributions

AZ developed the study design and wrote the first draft of the manuscript, KH, JV, and RV contributed in the testing and data analyses, SS and KW performed the statistical analysis.

### Conflict of interest statement

The authors declare that the research was conducted in the absence of any commercial or financial relationships that could be construed as a potential conflict of interest.

## Supplementary material

Supplementary Image 1 Forest plot with estimated effects (95% CI) of the confounders gender, ethnicity, BMI, PAQ score, and order of tests on balance performance. All confounders were adjusted for age.

Supplementary Image 2 Forest plot with estimated effects (95% CI) of the confounders gender, ethnicity, BMI, PAQ score, and order of tests on standing long jump performance. All confounders were adjusted for age.

Supplementary Image 3 Forest plot with estimated effects (95% CI) of the confounders gender, ethnicity, BMI, PAQ score, and order of tests on sprint performance. All confounders were adjusted for age.
