# Supplementary figures and images for: Motor Skills of Children and Adolescents Are Influenced by Growing up Barefoot or Shod
Source: Front Pediatr. 2018 Apr 25;6:115. doi: 10.3389/fped.2018.00115 (PMC5996942; doi:10.3389/fped.2018.00115)

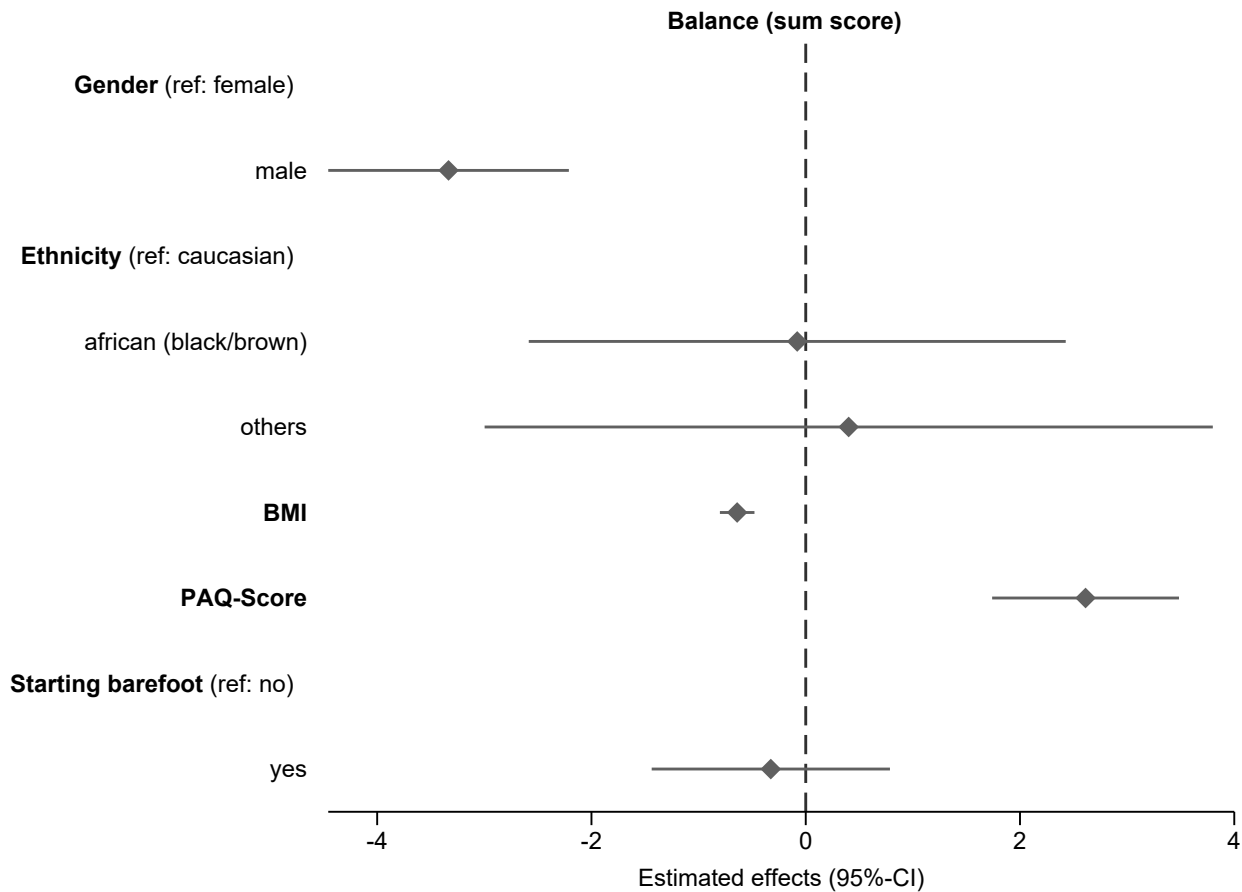

Supplement: Supplementary Image 1 — Forest plot with estimated effects (95% CI) of the confounders gender, ethnicity, BMI, PAQ score, and order of tests on balance performance. All confounders were adjusted for age. [file Image_1.pdf]

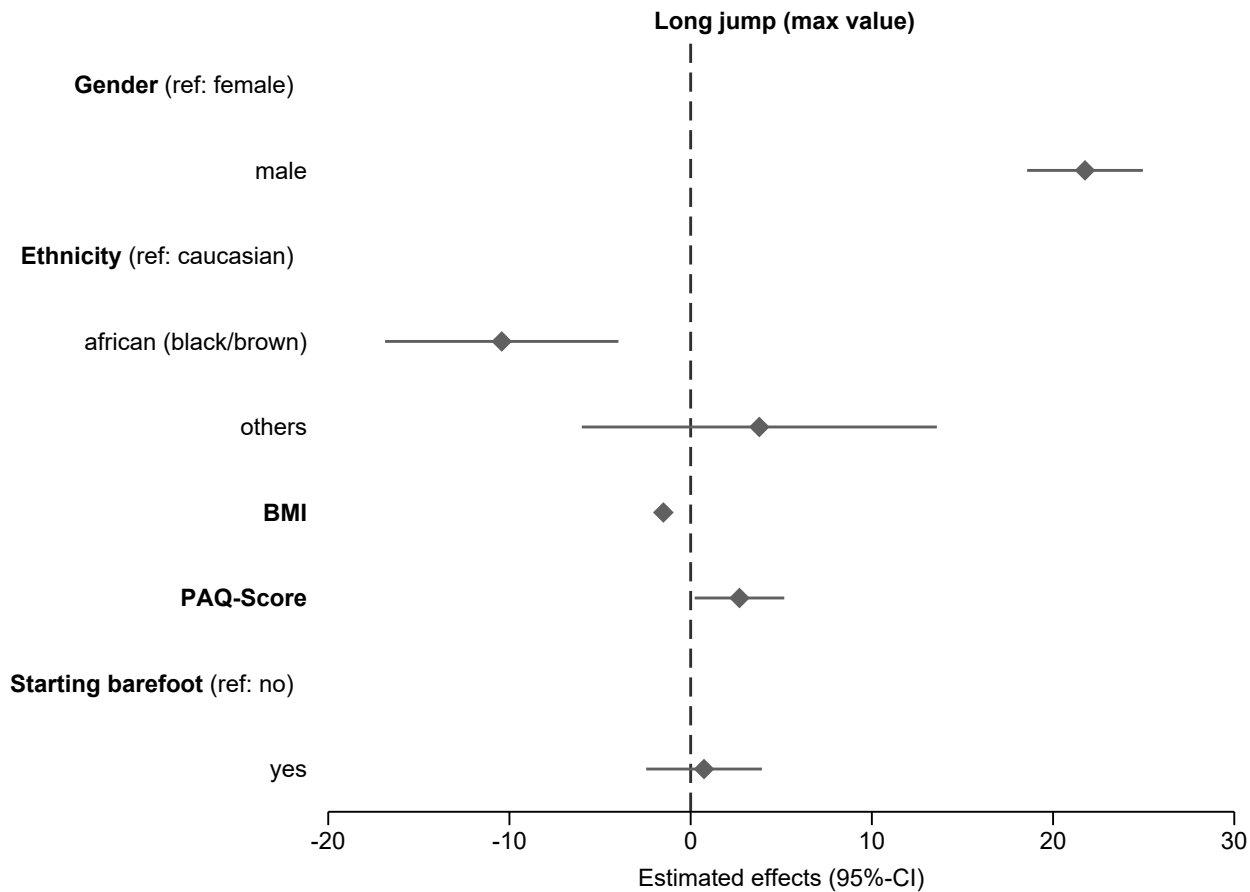

Supplement: Supplementary Image 2 — Forest plot with estimated effects (95% CI) of the confounders gender, ethnicity, BMI, PAQ score, and order of tests on standing long jump performance. All confounders were adjusted for age. [file Image_2.pdf]

# Sprint (max value within setting)

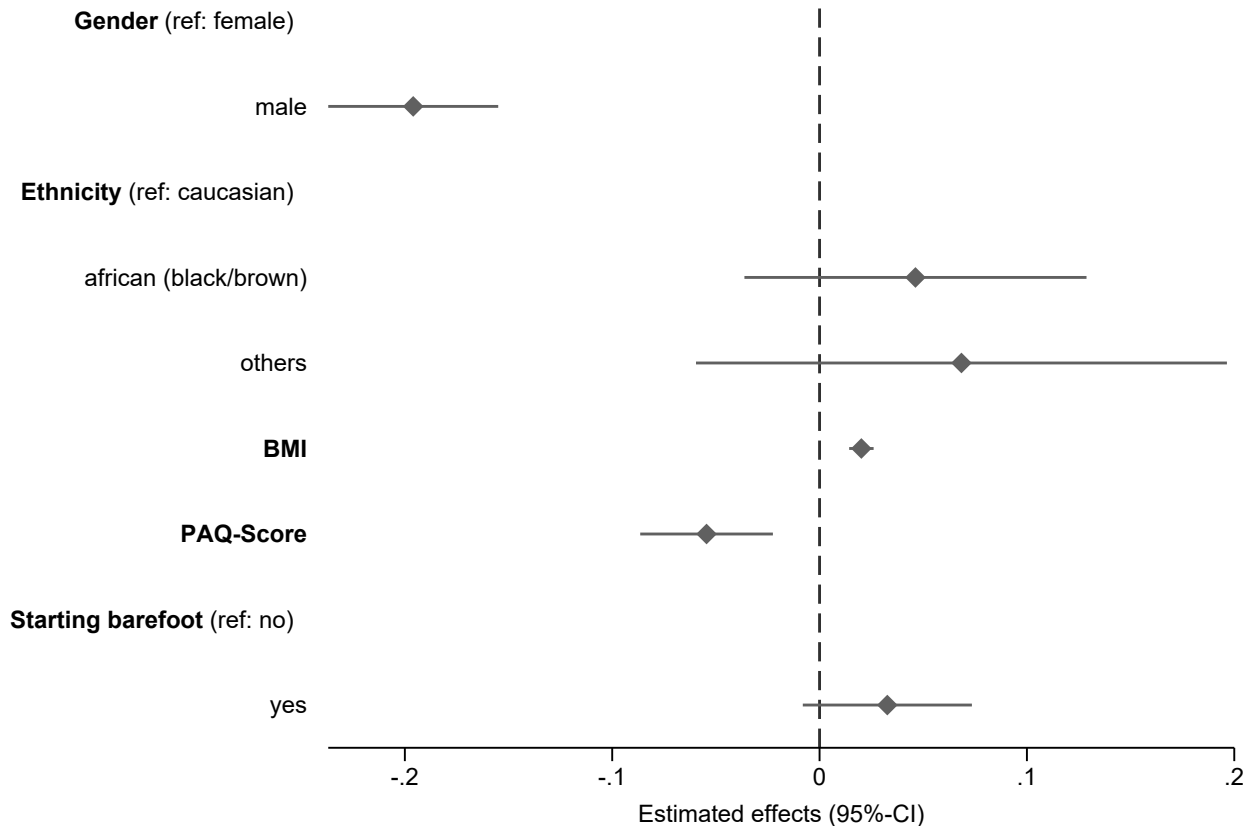

Supplement: Supplementary Image 3 — Forest plot with estimated effects (95% CI) of the confounders gender, ethnicity, BMI, PAQ score, and order of tests on sprint performance. All confounders were adjusted for age. [file Image_3.pdf]
